# Exploring Literature on Data Governance in the Health Care of Older Persons: Scoping Review

**DOI:** 10.2196/73625

**Published:** 2025-06-27

**Authors:** Angely Garcia, Ayra Mae Balingbing, Yves Palad

**Affiliations:** 1 Institute on Aging, National Institutes of Health, University of the Philippines Manila, 623 Pedro Gil St Ermita, Manila, 1000, Philippines; 2 College of Nursing, University of the Philippines Manila, Manila, Philippines; 3 College of Allied Medical Professions, University of the Philippines Manila, Manila, Philippines

**Keywords:** older persons, health, data governance, scoping review, medical records

## Abstract

**Background:**

Health data are growing rapidly, and the processing of such data is evolving. Research on data governance in older persons’ health care is unexplored, providing little guidance for practice and future studies.

**Objective:**

This scoping review aimed to synthesize available information on data governance in the context of older persons’ health based on evidence from literature.

**Methods:**

The study followed the methodological framework of Arksey and O’Malley and PRISMA-ScR (Preferred Reporting Items for Systematic Reviews and Meta-Analyses Extension for Scoping Reviews). Online databases, namely, PubMed, Cochrane, Ovid, ACM, IEEE Xplore, and Google Scholar were searched by 2 independent reviewers (AG and AMB) for studies on older persons’ health data governance published from January 2000 to April 2024. The independent reviewers performed the search, screening, data extraction, and review of full-text papers. A third reviewer (YP) made the final decision for unresolved discrepancies between the first 2 reviewers. The framework by the World Health Organization Pan American Health Organization, a high-level framework for planning and implementing data governance in public health, was used in the data extraction and analysis. Descriptive statistics were used, and a descriptive approach was used to summarize the results of the scoping review.

**Results:**

A total of 9840 titles were identified and 57 papers were included. Of these, 35 (61.4%) focused on technology, 19 (33.3%) on processes, and 3 (5.3%) on people. Data controller, processor, researchers, data subject or patient (including family or relatives), and relevant organizations were involved in older persons’ data governance. Data governance frameworks were designed and implemented by reviewing the current evidence, involving the stakeholders throughout the process, implementing specific procedures (eg, collection and aggregating health data), and monitoring and evaluating them.

**Conclusions:**

The review underscores the importance of the involvement of relevant stakeholders and the use of various innovative tools and approaches in governing data related to the health of older persons. Meanwhile, research specifically addressing data governance for older persons’ health conditions is limited. To enhance health outcomes for older persons, effective data governance is essential, alongside further research on relevant policies and practices.

## Introduction

Data governance is a management discipline and an emerging data management approach in health care. It is defined within the context of information technology as specifying the framework for decision rights and accountabilities to vitalize desirable behavior in the use of data [1]. Data governance highlights the responsibilities of those with authority in the organization [2], internal and external stakeholders [3], such as data stewards [4], civic society, public bodies [5], professional bodies [6], and the individuals who contributed their data [4], in managing data through its life cycle to generate quality information that can inform decision-making. Data life cycle begins with the capture or collection to the processing, use, storage, and disposal of data [7].

Data governance is also important in health systems as it is considered by many organizations as a promising method of maintaining health data [8]. Health data are considered an important asset to improve health through their use in public health, epidemiology, and health informatics [9]. Governance of such data allows health organizations to successfully manage, protect, maintain, and use data to generate information that improves health care quality, health outcomes, and health system performance [10].

Real-time generation and efficiency in obtaining knowledge are possible when data governance principles are applied in health care [3]. In contrast, the absence of data governance could lead to failure in decision-making and addressing the individual needs of the public sector [11]. Literature underscores the impact of data governance on a nation’s health care system, and the need to collect the right data, effectively process it to generate quality information for evaluating the health system, and identify where and when it is not functioning well [12]. Available evidence calls for the need to establish, streamline, and institutionalize a strong and comprehensive data governance process [5,13-19].

Data governance is essential in digital transformation initiatives of organizations as it improves data quality and accuracy and facilitates real-time data exchange [20]. One of the principles of the global health sector’s digital transformation is accelerating progress toward inclusive digital health with emphasis on the most vulnerable populations [15]. These vulnerable populations include the older persons. The potential of data governance in the field of older persons’ care has been highlighted by the World Economic Forum by presenting a new approach to data governance [21]. It emphasized that the combination of caregiver skills, for example, with the older persons’ data such as their specific care needs, can result in better and more precisely tailored care for older persons.

The state of research on data governance in the health care of older persons is unexplored locally and internationally. This leaves little guidance for its application in practice and for future research on the topic. Thus, this scoping review aimed to map out the available literature on data governance in the context of older persons’ health. Specifically, this review sought to answer the following questions: Who is involved in the planning and implementation of data governance (“people”)? How are data governance frameworks designed and implemented? What are the governance processes that lead to the improvement of older persons’ health (“process”)? What tools and technologies are used to effectively govern data (“technology”)?

## Methods

### Study Design and Framework

This scoping review followed the methodological framework of Arksey and O’Malley [22] and PRISMA-ScR (Preferred Reporting Items for Systematic Reviews and Meta-Analyses Extension for Scoping Reviews) [23]. The review questions were informed by the data governance framework in public health by Pan American Health Organization (PAHO), a high-level framework for planning, implementing, and continuously improving data governance [10]. The framework highlights the involvement of 3 components: people, processes, and technology. The people component includes establishing decision-making structures (eg, both the executive decision-making and the technical bodies); defining roles and responsibilities of those involved in the management of data throughout its life cycle; communicating these roles and responsibilities, data-related decisions, policies, and processes; and ensuring transparency on adherence to standards. Processes include managing data assets, enabling processes and standard operating procedures, and establishing processes for policy management and ensuring standards. The technology component pertains to identifying and implementing tools and technology required to manage data such as hardware and software; ensuring quality, availability, and security of information systems; and ensuring performance of the tools [10].

Aside from the framework for planning and implementing data governance, the data governance functions throughout the data life cycle also guided this scoping review. Data governance should address and include defining accountabilities, prioritizing investment requirements, establishing policies, implementing processes, setting standards, managing risks, and monitoring performance related to data throughout its life cycle. The data life cycle is as follows: data collection; data aggregation; data quality assurance and monitoring; data storage; data protection; data access, use, and disclosure; and data retention and destruction [10].

### Sources of Information

Six databases, namely, PubMed, Cochrane, Ovid, IEEE Xplore, Association for Computing Machinery (ACM), and Google Scholar were searched. PubMed, Ovid, and Cochrane contained a vast collection of health literature. IEEE Xplore and ACM digital libraries contained resource materials from the fields of electrical engineering, information technology, computer science, and electronics, which capture the technology-related aspect of this review. Google Scholar indexes the full text or metadata of scholarly literature and contains a wide variety of disciplines and sources.

### Search Strategy

The strategy was developed in consultation with an academic librarian and the search terms used were “data governance,” “health data governance,” “older person,” “older people,” “older adult,” “elderly,” “senior citizen,” and “aged.” Boolean operators were used to filter search results (Table S1 in Multimedia Appendix 1).

This review also included evidence from nonresearch sources [24] such as reports, projects, and economy papers from Google Scholar. Moreover, experts in data governance, eHealth, and geriatrics were consulted to explore additional literature sources. The experts were identified through the existing networks of the investigators and organizations relevant to digital health. Searching the reference list of the identified data governance literature was also conducted.

All the literature searched through the online databases, digital libraries, consultation with experts, and reference listing was uploaded in a shared Google Drive folder accessible only to the reviewers using their official university email addresses. The titles were encoded using Google Sheets to identify and remove duplicates.

### Selection Process

Considering that data governance was introduced in 2000 and literature on the topic started to be published around the same time [1,2,25], the search was filtered by publication dates between January 1, 2000, and April 22, 2024. The papers that meet the following criteria were included: (1) topic related to data governance including people, processes, or technology, (2) topic relevant to older persons’ health care, (3) peer-reviewed publications, reports, policies, programs, and policy briefs, and (4) in the English language. Resources that were not related to data governance and older persons’ health, conference abstracts, and published in languages other than English were excluded.

Following the eligibility criteria, 2 reviewers (AG and AMB) independently searched databases, screened papers for eligibility, reviewed the full texts, and extracted data from the included studies. If the reviewers are unable to screen papers based on the title alone, abstract screening was done to check whether the papers are related to health, data governance, and older persons. Initial data extraction from the abstracts was done to document the topic they covered using a data extraction form.

The reviewers independently documented the search yields and listed the titles obtained per online database using Google Sheets. The search yields of each reviewer per online database were compared as a form of initial validation. To ensure consistency among the reviewers, the procedures from searching to data extraction were pilot tested using 10 randomly selected samples. Reviewers proceeded with the next phases only upon reaching 75% agreement [24].

For each phase, the reviewers documented the reasons for exclusion and settled discrepancies by discussing them throughout the selection process. The reviewers met online and conferred about the discrepancies, which led to an amendment of the eligibility criteria. Based on the initial assessment of the abstracts and available full texts, there were various papers with mixed populations as their study samples (eg, 18 years and above). Considering the focus of this review, the reviewers decided to only include papers with older persons explicitly stated in their titles or abstracts or representing more than 50% of the study sample. Meetings between the reviewers were conducted to discuss which of the papers would be included in the review.

### Data Extraction

Once the list of included studies was finalized, the 2 reviewers (AG and AMB) independently extracted the relevant information using an extraction tool in Google Sheets. Information such as authors, citation, publication year, type of publication, research design, setting of care, population, data governance function and component, and outcomes of the study and the technological intervention, among others, were extracted for further analysis. The reviewers conducted regular meetings to discuss and reconcile differences in the extracted data. Any disagreements that remained unresolved were discussed with a third reviewer (YP) to arrive at a consensus. The third reviewer made the final decision for the unresolved disagreements between the first 2 reviewers.

### Data Management and Analysis

The search results and study selection process are documented in this scoping review report and presented using a PRISMA-ScR flow diagram. All data were documented in a Google Sheets document and exported into an Excel file after the entire process ended. For the unstructured texts in the data extraction form, a qualitative content analysis was conducted to describe how data governance is planned and implemented in the care of older persons. The data were coded according to the framework components involved, namely, person, processes, and technology, and data governance functions. A descriptive approach was used to summarize the result of the scoping review. Numerical or categorical data were presented using counts and proportions. Tables and a figure were used to present the extracted data for each extraction category.

### Ethical Considerations

The study was monitored and classified as exempt from ethics review (UPMREB 2023‐0864-EX) by the University of the Philippines Manila Research Ethics Board.

## Results

### Overview

**Figure 1.**
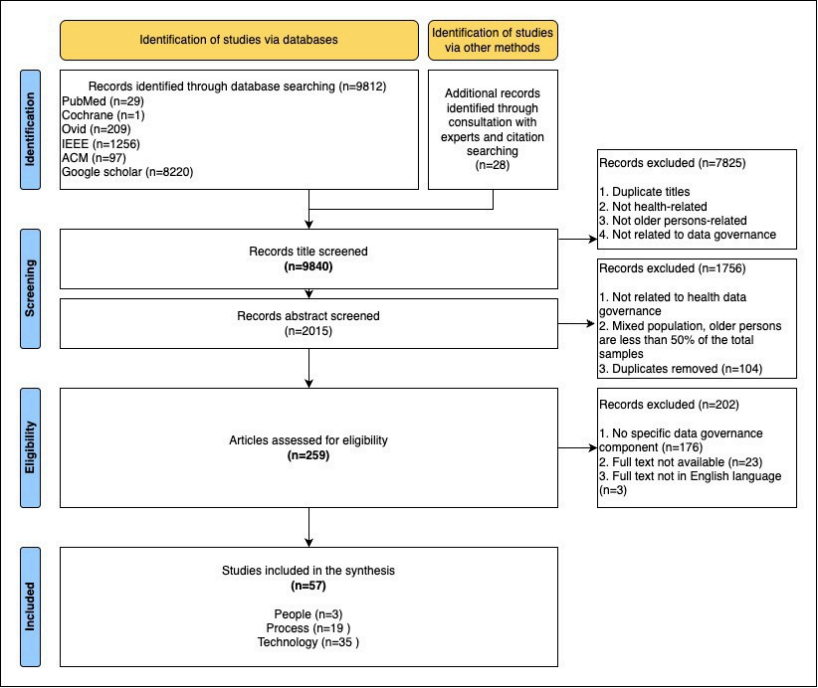
PRISMA-ScR (Preferred Reporting Items for Systematic Reviews and Meta-Analyses Extension for Scoping Reviews) diagram.

A total of 9840 titles were identified from 6 databases, consultation with 3 experts, and reference listing (Figure 1). Duplicates and all papers not related to older persons, health, and data governance, and those with mixed populations were removed during the screening phase, leaving a total of 259 papers assessed for eligibility. Of these papers, 202 were removed due to the unavailability of full texts, non-English language, and lack of specific data governance components. A total of 57 (22%) papers met the criteria for inclusion in the synthesis.

### Description of Included Studies

The included studies were published between 2003 and 2024; 56.1% (32/57) of these were published in the year 2020 onwards. The majority (32/57, 56.1%) of the included studies were from the IEEE digital library. Most (47/57, 82.5%) were journal papers, followed by conference proceedings (7/57, 12.3%). These conference proceedings were peer-reviewed full papers from IEEE and ACM (Table 1).

In terms of study design, 36.8% (21/57 ) were observational, followed by 29.8% (17/57) classified as experimental studies. These experimental studies are subdivided into 2 groups, namely, technology development and randomized controlled trials. About 30% (16/57 ) of the studies did not specify their design.

The studies were conducted mostly in home settings (11/57, 15.8%), followed by those conducted in multiple settings of care such as home and hospital, community and hospital, and hospital and long-term care facilities. More than 30% of the included studies did not specify their study setting. In terms of geographic coverage, most were conducted in Europe, followed by Asia and those with multicountry sites. There were no included studies that covered the following continents: Africa, Middle East, and South America (Table 1).

Sixty-seven percent of the included studies provided information about the study population, particularly, the age group, mean age, or age range they covered. Although 33.3% (19/57) did not indicate the specific age of their study population, the technology or the data governance component being relevant to older persons was explicitly stated in the title, abstract, and full text. Due to the variation of available information on age and unavailability of other information, the overall mean age cannot be computed.

About 70% of the included studies specified the sample size. A study had 1 older person sample, which was about a novel cloud-based framework for the older adult health care services [26]. The largest sample size among the included studies was 591,726 electronic health records of primary care patients aged 65 years and older [27].

**Table 1.** Profile of included studies.

Description	Included studies (n=57), n (%)	
Data sources		
IEEE	32 (56.1)	
Google Scholar	15 (26.3)	
ACM^a^	4 (7.0)	
PubMed	4 (7.0)	
Ovid	2 (3.5)	
Expert consultation	0 (0)	
Publication year		
2000‐2009	4 (7.0)	
2010‐2019	21 (36.8)	
2020 and onward	32 (56.1)	
Publication type		
Journal paper	47 (82.5)	
Conference proceeding	7 (12.3)	
Thesis	1 (1.8)	
Review	1 (1.8)	
Economy paper	1 (1.8)	
Study design		
Observational	21 (36.8)	
Experimental	17 (29.8)	
Technology development	15 (26.3)	
Randomized controlled trial	2 (3.5)	
Review (eg, systematic review and scoping review)	3 (5.3)	
Not specified	16 (28.1)	
Study settings		
Home	11 (19.3)	
Multiple settings	9 (15.8)	
Hospital	5 (8.8)	
Long-term care setting	5 (8.8)	
Community	4 (7.0)	
Others: research laboratory or special facility for experiment, university, office	4 (7.0)	
Not specified	19 (33.3)	
Geographic coverage		
Europe	12 (21.1)	
Asia	6 (10.5)	
Australia and Oceania	3 (5.3)	
North America	2 (3.5)	
Multicountry or continent sites	6 (10.5)	
Not specified	28 (49.1)	
Age (years) of older persons in study sample		
Specified (age covered/mean/range)	38 (66.7)	
Minimum age	18 (N/A^b^)	
Maximum age	104 (N/A)	
Not specified	19 (33.3)	
Sample size		
Specified	39 (68.4)	
Minimum sample size	1 (N/A)	
Maximum sample size (datasets)	591,726 (N/A)	
Not specified	18 (31.6)	

a ACM: Association for Computing Machinery.

b N/A: not applicable.

### Data Governance Components and Functions

A majority (35/57, 61.4%) of the included studies were largely related to technology, tools and technology, in particular (Table 2). This is followed by processes (19/57, 33.3%), which are mostly centered on enabling processes and standard operating procedures. Only 3 papers were focused on people*,* particularly, roles and responsibilities and decision-making structure.

Interestingly, there were no included studies on communication and transparency under the people component, standards and policy management in the process component, availability and security, and performance alone in the technology component (Table 2).

Table 2 also shows that the data governance function of the included studies mostly focused on implementing processes, while only a few covered monitoring performances [27-30], defining accountability [31-33], setting standards [34,35], and managing risks [36].

**Table 2.** Data governance components and functions of included studies.

Data governance	Included studies (n=57), n (%)	
Data governance component		
People	3 (5.3)	
None	54 (94.7)	
Roles and responsibilities	2 (3.5)	
Decision-making structure	1 (1.8)	
Communication and transparency	0 (0)	
Process	19 (33.3)	
None	37 (64.9)	
Enabling processes and SOPs^a^	11 (19.3)	
Data asset management	8 (15.8)	
Standards and policy management	0 (0)	
Technology	35 (61.4)	
None	22 (38.6)	
Tools and technology	33 (57.9)	
Tools and technology and performance	2 (3.5)	
Availability and security	0 (0)	
Performance	0 (0)	
Data governance function		
Implement processes	47 (82.5)	
Monitor performance	4 (7.0)	
Define accountability	3 (5.3)	
Set standards	2 (3.5)	
Manage risks	1 (1.8)	
Prioritize investment	0 (0)	
Establish policy	0 (0)	

a SOP: standard operating procedure.

### Designing and Implementing Data Governance

The identified actors involved in designing and implementing data governance related to the health of older persons include data controllers, processors, and subjects. Specific data controllers identified in this review include a hospital [32], researchers [33], and a steering committee [37]. The data subjects were patients [32], persons with dementia [33], nursing home residents [37], friends or relatives [32], legally authorized representatives [33], and hospital staff [32]. The data processors were a platform service provider [32], research team members [33], and privacy, scientific expert, and data access committees [37].

All included studies on the people component of data governance highlighted the crucial roles of relevant stakeholders (eg, controllers, processors, and subjects) in planning, designing, and implementing data governance [27,32,33]. Moreover, the composition, roles, and accountabilities of these stakeholders vary depending on the settings (eg, hospital, research, and nursing home).

An example specifying the roles and responsibilities of people involved in data governance was highlighted by Bernsmed [32] in the context of medical data collected from sensors that are exchanged between the older persons, their families and friends, and health personnel. The roles of data controller, processor, and subject were defined, and the accountability obligations were outlined. A data controller, which can be a person, public authority, agency, or other actors, determines the purposes and means of personal data processing. The data processor processes the personal data on behalf of the controller. Meanwhile, a data subject is a person who can be identified directly or indirectly through a reference identification number. In the study, the patients and relatives or friends, as well as the hospital staff, were the data subjects, while the hospital is the controller of its patients’ and personnel’s data. The data controller (hospital) is accountable to patients, relatives or friends, and hospital staff (data subjects) in informing and obtaining consent for the collection, processing, and management of their data [32].

A total of 10 papers provided information on how frameworks related to data governance of older persons’ health data are designed and implemented [26,32,33,35,37-42]. Specific examples of the use of data governance frameworks were related to addressing a particular issue such as fall detection [41], dementia care mapping [42], depression risk prediction [35], cardiac image processing [40], and digital twin health care for real-time supervision and crisis warning [43].

Conceptual content analysis of the methods using the PAHO framework emphasized the involvement of relevant stakeholders, implementation of specific processes, and conduct of literature review, demonstrating the feasibility or testing of the developed framework and defining the roles and accountabilities of the actors or stakeholders (Table S2 in Multimedia Appendix 1).

### Data Governance Processes, Tools, and Outcomes

A total of 19 papers provided information on the specific processes or interventions relevant to older persons’ health [26,30,34,35,38,39,41-53]. Majority focused on implementing processes pertaining to data collection, aggregation, and access, use, and disclosure. One study covered 6 data life cycles from data collection up to disclosure [44]. There were no papers that covered the process of defining accountability, managing risks, prioritizing investment, and establishing policy (Table 2).

Several tools and technologies were identified and used for data governance throughout the data life cycle. A total of 35 papers provided information on the specific technologies relevant to older persons’ health [27-29,36,40,54-82]. Most of the identified technologies focused on implementing processes while only a few were related to use of technology in monitoring performance, defining accountability, and managing risks. Furthermore, these technologies are mostly involved in data collection.

In implementing data governance processes, various tools and technologies are used in collecting data for the assessment and monitoring of health needs; assessment of fall risk and detection of fall; identification of mental health–related issues such as mild cognitive impairment, dementia, stress, loneliness, and depression; and improvement of lifestyle and health (Table S2 in Multimedia Appendix 1).

In terms of specific outcomes of the technology component of data governance, a total of 34 papers provided information on these. Most of the outcomes were process-related, such as efficient data access; quality control; communication and care support; recognition, detection, and prediction of various conditions; monitoring; and others. Only 2 included papers provided information on health outcomes among the older person samples, namely, improvement of quality of life [62] and cognitive skills and function [68] (Table S2 in Multimedia Appendix 1).

### Older Persons’ Health Care Data Governance

**Figure 2.**
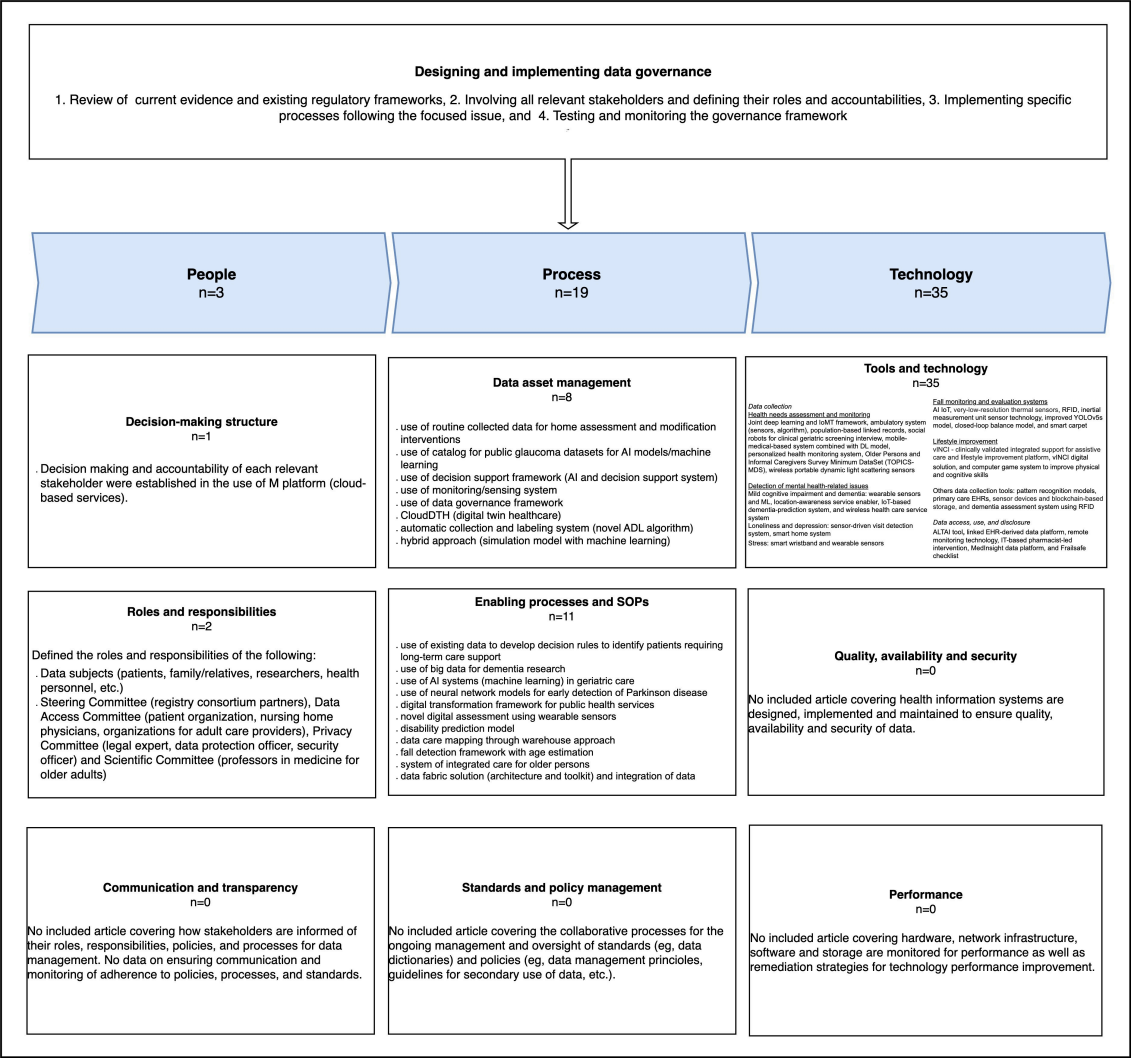
Older person’s health care data governance based on the available literature. ADL: activities of daily living; AI: artificial intelligence; EHR: electronic health record; IoT: Internet of Things; DL: deep learning; ML: machine learning; RFID: radio frequency identification; SOP: standard operating procedure.

Figure 2 summarizes the results of this scoping review following the data governance framework in public health by PAHO [10].

## Discussion

### Principal Results

This scoping review aimed to synthesize available information on how data governance is planned and implemented in the context of older persons’ health based on evidence from literature. Specifically, it aimed to answer the following questions: Who are involved in the planning and implementation of data governance (people)? How are data governance frameworks designed and implemented? What are the governance processes that lead to the improvement of older persons’ health (process)? What tools and technologies are used to effectively govern data (technology)?

Findings show that available studies related to the health of older persons covered mostly data governance components related to technology and process. Moreover, data governance is designed and implemented by initially examining the current evidence and existing regulatory frameworks, involving all relevant stakeholders and defining their roles and accountabilities, implementing specific processes following the focused issue, and testing and monitoring the governance framework. Various data governance processes, tools, and technologies are used and contribute to the improvement of older persons’ health. The use of these mostly leads to process outcomes.

This review emphasizes the involvement of stakeholders, particularly in technology development and testing process [40,41], sharing perspectives on digital transformation of public health services [39], and mapping of service users [42]. Moreover, the accountability of data controllers, processors, and subjects was outlined [32,33,37]. This underscores the data governance responsibilities of those with the authority in the organization [2] (data controller), the internal and external stakeholders (data processors and subjects) [3-6], and, ultimately, the data subjects who contributed their data [4] in managing data through their life cycle to inform decision-making.

### Limitations

This review included literature only on data governance relevant to older persons published from the past 2 decades and those peer-reviewed publications, reports, policies, programs, or policy briefs written in English language. Identified studies eligible for review but without full texts were excluded from the synthesis. Despite the efforts of the reviewers and consultant librarian in accessing the papers, 23 full texts were not retrieved. These papers were mostly from ACM and Google Scholar, published 2021 and beyond, included conference proceedings, and focused on the technology component of data governance. These exclusions might have affected the comprehensiveness of the reported findings on technology.

Many included studies did not specify the study design, setting, geographic coverage, age of the study population, and sample size. It would be helpful if this information were available to provide a complete description of the state of data governance literature for older persons. Moreover, the assessment or appraisal of the included studies for methodological rigor and quality was not performed as this review aimed to describe information only on data governance in the context of older persons’ health.

Various processes and technologies aimed to contribute to the improvement of older persons’ health based on their objectives and purpose were identified. However, since most of the included studies are related to technology, at their preclinical stage of technological development, and focused only on data collection, limited information on the actual health outcomes of data governance specific to older persons was collected.

### Comparison With Prior Work

Studies related to health information systems included in this review [27,60,79] also support data governance as an important player in the health system, a promising method of maintaining health data [8], and recognize its potential in the field of older persons’ care [83,84]. Various processes, tools, and innovative approaches in governing health data of older persons were explored in this review. Available evidence suggests that the use of these tools and technologies supports data governance, leading to positive health outcomes and better processes [27,31,61-63,68,75-77]. Although positive health outcomes were noted, further interventional studies with utilization of a data governance framework are essential to determine its direct effects on actual health outcomes among the older population.

In terms of processes, findings in this review are consistent with Cave [85] where strategies for implementing data governance were explored through a qualitative case study. These strategies include structured oversight with committees and boards, obtaining stakeholder buy-in, and benchmarking and standardization. Benchmarking and standardization through review of current evidence and existing regulatory frameworks were identified to be important steps in planning and implementing data governance [32,33,35,38,39,42]. These preliminary steps are crucial in ensuring a well-designed and effective governance framework.

Strategies for effective and strategic communications and compliance with regulations were not adequately covered in this review due to the lack of studies on this topic. Communication and transparency, as well as compliance with standards and policies, should be considered in implementing data governance. This is to ensure the protection of people, promotion of health value, and prioritization of equity, which are the key health data governance principles [86]. Moreover, although there were no papers that covered the processes of defining accountability and managing risks, these were discussed in the literature in terms of the role of persons involved in data governance and as integral components of designing and implementing data governance.

Various geriatric syndromes, such as falls and fall risk [54,55,65,69,71,73,74], mild cognitive impairment and dementia [33,42,53,64,66,75,82], frailty [29,80], loneliness, social isolation, and depression [56,57], were the focus of the studies included in this review. However, there were no included studies related to other geriatric syndromes such as functional decline, incontinence, delirium, pressure ulcers, polypharmacy, malnutrition, sleep problems, and others. These geriatric syndromes are common health conditions among older persons [87-90], often having multifactorial causes, and may have a major impact on their quality of life and disability [90]. Data governance covering these conditions can also generate information on strategies to address the needs of older persons and provide quality care to improve their health and quality of life.

Only 1 paper explicitly proposed a data governance framework specific for older persons, which was published a decade ago [38]. The proposed framework was drawn from the Data Management Body of Knowledge of the DAMA International, Inc [2,91] and the work of Cleven and Wortmann [92]. Dahlberg’s [38] motive for proposing this specific governance framework was the fragmentation of older persons’ data and the necessity to consolidate these data to make it more useful. The paper defined the types and sources of data that exist about older persons, and how these can be integrated into a comprehensive framework. It suggested defining the data categories, attributes, and sources of data first to improve data governance of older persons. There is no evidence that the framework has been validated. Hence, testing this framework is essential to determine its value in data governance for older persons.

It is notable that the included papers that provided information on designing and implementing data governance mostly came from high-income countries such as Australia, United States, Canada, United Kingdom, Japan, Singapore, Netherlands, and other countries in Europe. These countries are actively implementing digital health care solutions in the forms of telemedicine, electronic records, mobile health, and other digital tools for diagnosis and management, hence, the availability of data governance frameworks. On the other hand, no studies were included in the review from Africa, Middle East, and South America. This can be attributed to the challenges experienced in digital health, such as limited infrastructure, affordability issues, and data privacy concerns, especially in low-income and middle-income countries [93-95]. While others have established and are implementing data governance, the rest are yet to address the challenges of digital health transformation.

The need for data governance in general [14] and in the care of older persons has been highlighted by the reviewed papers specifically on dementia research [42,53], health care applications for geriatric clinical care [49], and nursing homes for quality improvement [37]. New paradigms on the use of big data in dementia research and clinical care [42], as well as international dementia care mapping, require data governance [53]. Likewise, the standardization of machine learning approaches tailored to health care applications is required to evaluate whether these applications improve clinical care for older persons [49]. In nursing homes, literature suggests the need for enhancing transparency, specifically in presenting understandable information to the residents and their representatives on which data will be used, how they will be used, and for what purposes [37].

Findings agree with other papers not included in this review such as the call for institutionalizing a strong and comprehensive data governance process [5,10,12-19,96-98], a national health data governance framework to ensure availability and use of personal health data for public interest [99], and data governance for learning health systems to reduce concerns about privacy and trust in the system [100,101].The included studies were analyzed according to the data life cycle, data governance function, and components they covered to identify research gaps. The people data governance component covered only data access, use, and disclosure. Information on how people are involved, their roles and responsibilities, and communication and transparency during data collection, aggregation process, quality assurance, storage, data protection, retention, and destruction, is lacking. In terms of the process component, limited studies were related to data quality, storage, and protection, and none for defining accountability and managing risk. Meanwhile, no tools and technology were identified from the studies that covered data aggregation, data quality, data protection, and setting standards.

Studies on data governance in health care of older persons are largely focused on various forms of technology followed by process. The major research gap across all data governance components was the lack of included studies on data retention and destruction, prioritizing investments, and establishing policy (Table S3 in Multimedia Appendix 1). Findings and gaps that were identified from this review inform future research directions and practices of older persons’ health data governance.

### Conclusions

This review highlighted the importance of benchmarking, involvement of relevant stakeholders, and the use of various innovative tools and approaches in governing data related to the health of older persons. However, studies that are explicitly centered on data governance of older persons’ health data, their common health conditions, or other geriatric syndromes, are limited. Likewise, identified research gaps include the lack of studies on data retention and destruction, establishment of policy, prioritizing investment, and communication and transparency.

Available studies on the health of older persons covered mostly technology and process data governance components. Only a few focused on people*,* which were identified to have crucial roles in designing governance frameworks, planning, and implementing data governance. To optimize the use of data in improving the health and quality of life of older persons, a well-designed process that considers the essential data governance components, functions, and principles should be developed and implemented.

### Recommendations

Effective governance of older persons’ health data requires a multisectoral approach. Benchmarking, utilization of innovative tools and approaches, and collaboration between relevant stakeholders, including policy makers, program planners, health facility administrators, health care providers, information and communication technology and digital health experts, data privacy and legal experts, and, ultimately, the older persons and their families, are essential in designing and implementing data governance.

In establishing health data governance policies or incorporating them into the national standards, legislations, and organizational policies, the key governance principles, components, functions, and data life cycle should be considered. In practice, health settings and personnel should ensure the involvement of the patients (including their families and relatives) in collecting and processing their data. The practical application of data governance frameworks and technologies in the care of older persons is essential to support favorable health outcomes. Further studies on the governance of data on common health conditions of older persons to inform clinical practice, health data retention schedules and destruction methods, and data governance policies and investments are recommended.

## Supplementary material

10.2196/73625 Multimedia Appendix 1 Search terms used by database, summary of extracted entries from the included studies, and research gaps on data governance in the health care of older persons.

10.2196/73625 Checklist 1 PRISMA-ScR (Preferred Reporting Items for Systematic Reviews and Meta-Analyses Extension for Scoping Reviews) checklist.

## Abbreviations

ACM: Association for Computing Machinery
PAHO: Pan American Health Organization
PRISMA-ScR: Preferred Reporting Items for Systematic Reviews and Meta-Analyses Extension for Scoping Reviews
