# Supplementary material for: Exploring Literature on Data Governance in the Health Care of Older Persons: Scoping Review
Source: JMIR Aging. 2025 Jun 27;8:e73625. doi: 10.2196/73625 (PMC12227155; doi:10.2196/73625)
Supplement: Multimedia Appendix 1 [file aging-v8-e73625-s001.docx]

Table S1. Search terms used by database.

| Database | Search terms |
| --- | --- |
| Pubmed | ("older person" OR "older people" OR "older adults" OR "senior citizens" OR "seniors" OR "elderly" OR "aged") AND ("data governance" OR "health data governance") |
| IEEE Xplore | (((("Abstract":"older person") OR ("Abstract":"older people") OR ("Abstract":"older adults") OR ("Abstract":"senior citizens") OR ("Abstract":"seniors") OR ("Abstract":"elderly") OR ("Abstract":"aged") AND ("Abstract":"data governance") OR ("Abstract":"health data governance"))) ) |
| ACM | Fulltext:("older person" OR "older people" OR "older adults" OR "senior citizens" OR "seniors" OR "elderly" OR "aged") AND Fulltext:("data governance" OR "health data governance") |
| Cochrane | ((("older person"):ab OR ("older people"):ab OR ("older adults"):ab OR ("senior citizens"):ab OR ("seniors"):ab OR ("elderly"):ab OR ("aged"):ab) AND (("data governance"):ab OR ("health data governance"):ab)) |
| Ovid | (FullText:("older person" OR "older people" OR "older adults" OR "senior citizens" OR "seniors" OR "elderly" OR "aged") AND ("data governance" OR "health data governance")) |
| Google Scholar | ("older person" OR "older people" OR "older adults" OR "senior citizens" OR "seniors" OR "elderly" OR "aged") AND ("data governance" OR "health data governance") |

Table S2. Summary of extracted entries from the included studies.

| **Title** | **Author/s**  **Year** | **Research design** | **Population** | **Intervention** | **Outcome of the Study** | **Outcome of the Technological intervention** | **Data Governance Life-cycle** | **Data Governance Function** |  |
| --- | --- | --- | --- | --- | --- | --- | --- | --- | --- |
| **Technology (n=35)** | | | | | | | | |  |
| A Feasible Fall Evaluation System via Artificial Intelligence Gesture Detection of Gait and Balance for Sub-Healthy Community- Dwelling Older Adults in Taiwan | Lin & Wai, 2021 | Not specified | 65+ | fall evaluation system AI IoT | TUGT and grip strength measures, database, sarcopenia prevention/alert system | gait speed | data collection | implement processes |  |
| A Healthcare Integration System for Disease Assessment and Safety Monitoring of Dementia Patients | Lin et al., 2008 | Observational (cross sectional) | Not specified | dementia assessment system & radio frequency identification (RFID) | dementia assessment system | patient's general location | data collection | manage risks |  |
| A Joint Deep Learning and Internet of Medical Things Driven Framework for Elderly Patients | Zhang et al., 2020 | Observational (cross sectional) | Not specified | joint deep learning and Internet of Medical Things framework | Not specified | Not specified | data collection | implement processes |  |
| A Nonintrusive Elderly Home Monitoring System | Fang et al., 2021 | Observational (cross sectional) | Not specified | sensor device, open-hardware software platform, data encryption, one-class support vector machine | reliability, accuracy, and efficiency of the system | continuous monitoring, data privacy preservation, efficient data access and control | data collection | define accountability |  |
| A Novel Monitoring System for Fall Detection in Older People | Taramasco et al., 2018 | Not specified | Not specified | fall detection system | accuracy of fall detection | fall detection | data collection | implement processes |  |
| A Sensor-Driven Visit Detection System in Older Adults’ Homes: Towards Digital Late-Life Depression Marker Extraction | Schutz et al., 2022 | Observational (cohort) | 86± 7.23, | sensor-driven visit detection system with various approaches | visit detection, correlation with GDS score | home visits | data collection | implement processes |  |
| A Smart-Home System to Unobtrusively and Continuously Assess Loneliness in Older Adults | Austin et al., 2016 | Observational (longitudinal) | 62-80 (71.0) | smart home system (sensors, phone monitors, computer software) | loneliness | in-home mobility, walking speed, time spent on computer, time spent on phone, number of phone calls, time out of home, number of computer sessions | data collection | implement processes |  |
| ALTAI Tool for Assessing AI-Based Technologies: Lessons Learned and Recommendations from SHAPES Pilots | Rajamäki et al., 2023 | Observational (cross sectional) | 65+ | Assessment List for Trustworthy AI (ALTAI), an interactive self-assessment tool | concerns on AI-based technologies, list of recommendations | list of recommendations | data access, use, and disclosure | implement processes |  |
| Ambulatory system for human motion analysis using a kinematic sensor: monitoring of daily physical activity in the elderly | Najafi et al., 2003 | Experimental | 65+ | ambulatory system (sensors, algorithm) | sensitivity, specificity, accuracy, detection errors | body posture detection/postural; transitions (lying, sitting, standing, walking); | data collection | implement processes |  |
| Biomechanical Parameters and Clinical Assessment Scores for Identifying Elderly Fallers Based on Balance and Dynamic Tasks | Pradhan et al., 2020 | Experimental | 65 and above; 58 non-fallers (72.3 SD 5.7), 41 fallers (74 SD 12.3) | pattern recognition models (clinical assessment scores vs biomechanical parameters vs ML models) | accuracy for identifying fallers/NF, specificity, sensitivity | Ground reaction force, center of pressure, plantar pressure | data collection | monitor performance |  |
| Characterising complex health needs and the use of preventive therapies in the older population: a population-based cohort analysis of UK primary care and … | Elhussein et al., 2023 | Cohort study | 65 and above | population-based linked records | cohort identification & characteristics | comorbidities, prevalence & incidence of preventive therapy use | data collection | implement processes |  |
| Clinical Screening Interview Using a Social Robot for Geriatric Care | Manh Do et al., 2021 | Not specified | 60-89 | clinical screening interview through social robots | comprehensive clinical screening (cognitive assessment, fall risk evaluation, pain rating), general clinical interview management | conversational interface, face tracking, an interaction handler, attention management, robot skills, and cloud service management. | data collection | implement processes |  |
| Clinically-validated technologies for assisted living: The vINCI project | Spinsante et al., 2023 | Pilot study | 58-94 (73.05) mixed population | assistive technologies - vINCI | user needs perspectives, preferences, requirements | evaluation of physical activity, improve QOL | data collection | implement processes |  |
| Clinically-validated technologies for older adults' quality of life self-management: vINCI ecosystem | Băjenar et al., 2022 | Experimental | 65 and above | vINCI technology (personalized digital solution) | Not specified | quality control, data governance and automatic monitoring | data collection | implement processes |  |
| Cognitive Training and Stress Detection in MCI Frail Older People Through Wearable Sensors and Machine Learning | Delmastro et al., 2020 | Randomized cross over observational study | 65+ years old | wearable sensors and machine learning | pilot study, stress response, evaluation of stress detection system, mobile system architecture for stress monitoring, | stress detection | data collection | implement processes |  |
| Compressive Representation for Device-Free Activity Recognition with Passive RFID Signal Strength | Yao et al., 2018 | Experimental | Not specified | RFID-based activity recognition system | system performance (accuracy) | activity recognition dictionary learning | data collection | implement processes |  |
| Design and Technical Evaluation of an Enhanced Location-Awareness Service Enabler for Spatial Disorientation Management of Elderly With Mild Cognitive Impairment | Moreno et al., 2015 | Experimental | Not specified | location-awareness service enabler | feasibility, usability | location, wandering episodes detection | data collection | implement processes |  |
| Developing a linked electronic health record derived data platform to support research into healthy ageing | Andrew et al.,  2023 | Not specified | 60+ years and above | linked EHR derived data platform | development of EHR based research data warehouse | better understanding on the complexity of ageing | data access, use, and disclosure | implement processes |  |
| Development and Evaluation of a Computer Game Combining Physical and Cognitive Activities for the Elderly | Lin et al., 2020 | Historical control study design | 65 years and older | computer game system | cognitive function, motor function, game evaluation (short term memory, divided attention, inhibitory function), and user's feedback (willingness to use, difficulty levels of games) | improvement in cognitive skills, cognitive function | data collection | implement processes |  |
| Development of Data-Driven Metrics for Balance Impairment and Fall Risk Assessment in Older Adults | McManus et al., 2022 | Experimental | 71.48±7.35 years 74.9±6.5 years | inertial sensor technology (IMU)-based balance assessments | accuracy, test reliability | balance and fall risk measurements (balance score and weighted balance score) | data collection | implement processes |  |
| Effective Data Decision-Making and Transmission System Based on Mobile Health for Chronic Disease Management in the Elderly | Wu et al., 2021 | Experimental | 65 years and above | mobile-medical-based system; deep learning model-combined sparse autoencoder (CSAE) | accuracy, sensitivity, and specificity. | classification and prediction of chronic diseases | data collection | implement processes |  |
| Elderly Fall Detection Based on Improved YOLOv5s Network | Chen et al., 2022 | Ablation experiment | Not specified | improved YOLOv5s model for fall detection | average accuracy of the algorithm | falling behavior | data collection | implement processes |  |
| Frequency and impact of medication reviews for people aged 65 years or above in UK primary care: an observational study using electronic health records | Joseph et al., 2023 | Observational | OP 65 years and over | primary care EHRs | medication reviews, prescribed medicines and prescription count, factors associated with med review (care home residence, previous med review, baseline rx count) | medication reviews, prescribed medicines and prescription count, factors associated with med review (care home residence, previous med review, baseline rx count) | data collection | monitor performance |  |
| Healthcare professionals views on technology to support older adults transitioning from hospital to home | Doyle et al., 2016 | Not specified | 64-94 (mean 83) | remote monitoring technology for transition care | potential barriers and facilitators to integration within HS, roles for technology to support transitional care | transitional care support | data access, use, and disclosure | implement processes |  |
| Home-Based Risk of Falling Assessment Test Using a Closed-Loop Balance Model | Ayena et al., 2015 | Experimental | 68.4+-5.5 (healthy); 66.28+-8.9 (PD) | closed-loop balance model | real time computation of risk of falling in daily activities | one-leg standing test (OLST) score and risk of falling | data collection | implement processes |  |
| Improving patient safety for older people in acute admissions : implementation of the Frailsafe checklist in 12 hospitals across the UK | Papoutsi et al., 2018 | mixed methods | Not specified | Frailsafe checklist; reporting of high-level process data (completion of checklist and relevant frailty assessments). | Volume and patterns of checklist use, perceived usefulness, multidisciplinary working, reliability of frailty assessments | increase completion of key clinical assessments, facilitate communication for the care of OP | data storage | monitor performance |  |
| In-Home Floor Based Sensor System-Smart Carpet- to Facilitate Healthy Aging in Place (AIP) | Muheidat et al., 2020 | Not specified | Not specified | smart carpet (Context-aware and private real-time reporting aging in place system) | sensitivity, specificity, accuracy, error difference in walking speed, average accuracy in predicting fall risk and counting people | fall detection, gait measurement, sociability (#of people traversing the carpet) | data collection | implement processes |  |
| IoT-Based Unobtrusive Physical Activity Monitoring System for Predicting Dementia | Kim et al., 2022 | Experimental | 65-79 | IoT-based dementia-prediction system for monitoring physical activities | accuracy | physical activity monitoring, dementia risk prediction | data collection | implement processes |  |
| Personalized Health Monitoring System of Elderly Wellness at the Community Level in Hong Kong | Yu et al., 2018 | Not specified | 65+ years old | personalized health monitoring system | classification accuracy, recall, precision, F-score | health monitoring | data collection | implement processes |  |
| Preventing a rise in anticholinergic and sedative medication load: Feasibility of an innovative pharmacist-led intervention [Feasibility, acceptability and potential effectiveness of an information technology- based, pharmacist-led intervention to prevent an increase in anticholinergic and sedative load among older community-dwelling individuals] | Van der Meer et al., 2019 | prospective study | 76.5 (8.0) | IT-based pharmacist-led intervention | feasibility, acceptability and potential effectiveness | rational prescribing | 6 | implement processes |  |
| Smart Wristband-Based Stress Detection Framework for Older Adults With Cortisol as Stress Biomarker | Nath & Thapliyal, 2021 | Experimental | 73.625 ± 5.39. | smart wristband | F1-score, accuracy | stress detection (from biomarkers) | data collection | implement processes |  |
| The development of the Older Persons and Informal Caregivers Survey Minimum DataSet (TOPICS-MDS): a large-scale data sharing initiative | Lutomski et al., 2013 | Not specified | 41 research projects (32,310 OPs, 3,940 caregivers) | dataset (TOPICS-MDS, data sharing initiative) | characteristics of projects | data repository (with essential data to better understand health challenges of OPs and caregivers) | data collection | implement processes |  |
| The feasibility of deriving the electronic frailty index from Australian general practice records | Lewis et al., 2022 | Retrospective exploratory | 80 years (SD 6.5) | data platform (MedInsight) + set of rules | frailty prevalence, eFI score | presence of frailty | data access, use, and disclosure | implement processes |  |
| Wireless Dynamic Light Scattering Sensors Detect Microvascular Changes Associated With Ageing and Diabetes | Zherebtsov et al., 2023 | Experimental | 60+-12; 58+-12; | wire- less portable dynamic light scattering sensors | accuracy | microvascular changes detection (thru blood perfusion recordings) | data collection | implement processes |  |
| Wireless Health Care Service System for Elderly With Dementia | Lin et al., 2006 | Experimental | Not specified | Wireless Health Care Service System | system performance (system response time, transmission efficiency, location accuracy), user satisfaction | satellite positioning, wireless communication, and information processing | data collection | implement processes |  |
| **Process (n=19)** | | | | | | | | |  |
| A Catalog of Public Glaucoma Datasets for Machine Learning Applications: A detailed description and analysis of public glaucoma datasets available to machine learning engineers tackling glaucoma-related problems using retinal fundus images and OCT images. | Kiefer et al., 2023 | Observational | 62.6; 55.2' 35-80; 18-76 | catalog for public glaucoma datasets for AI models/machine learning | standardized datasets (comprehensive glaucoma dataset catalog) | Not specified | data aggregation | set standards |  |
| A Decision-Support Framework for Promoting Independent Living and Ageing Well | Billis et al.,  2015 | Observational (cross sectional) | Not specified | decision support framework (artificial intelligence and decision support system) | Sleep problems, depression diagnosis, depression severity, risk of future progression to depression | trend analysis, decision support core, risk prediction and assessment | data aggregation | implement processes |  |
| A framework for and issues in the management and governance of data on elderly citizens (ongoing res.) | Dahlberg, 2014 | Observational (cross sectional) | Not specified | data governance framework | governance framework for elderly | Not specified | data collection | set standards |  |
| A Novel Cloud-Based Framework for the Elderly Healthcare Services Using Digital Twin | Liu et al., 2019 | Observational (cross sectional) | Not specified | CloudDTH (digital twin healthcare) | framework and application methods of digital twin healthcare in the cloud | monitoring, diagnosing, and predicting aspects of health of individuals | data collection | implement processes |  |
| A Novel Fall Detection Framework with Age Estimation Based on Cloud-fog Computing Architecture | Lin et al., 2024 | Not specified | pedestrians aged 60 and above | fall detection framework with age estimation | accuracy, detection speed,. mean absolute error, miss rate | fall detection, age estimates | data collection | implement processes |  |
| A system of integrated care for older persons with disabilities in Canada: results from a randomized controlled trial | Béland et al., 2006 | RCT | 64-104 | System of integrated care for OP | utilization, public costs of institutional and community care; health status, satisfaction with care, caregiver burden, out of pocket expenses | None | data collection | implement processes |  |
| A Versatile Data Fabric for Advanced IoT-Based Remote Health Monitoring | Buleje et al., 2023 | Not specified | 70+ | data fabric solution (architecture and toolkit) - integration of data | data fabric architecture, effectiveness of data fabric solution | cognitive, social, and mobility impacts of clinical and subclinical sleep disturbance (e.g., sleep apnea) | data collection  data aggregation  data quality  data storage  data protection  data access, use, and disclosure | implement processes |  |
| Anticipating care needs of patients after discharge from hospital: frail and elderly patients without physiological abnormality on day of admission are more likely to require social services input. | Subbe et al., 2017 | Not specified | 80.4 (SD 9.9), 67.5 (14.2) | Fy Nhaid- service evaluation (use of existing data to develop decision rules to identify patients requiring LTC support) | clinical frailty scale, visit number (number of admissions), simple clinical score, National Early Warning Score (NEWS) | social services requirement | data access, use, and disclosure | implement processes |  |
| Behavioral Patterns of Supply and Demand Sides of Health Services for the Elderly in Sustainable Digital Transformation: A Mixed Methods Study | Zhou et al., 2022 | Mixed methods | mixed population (59.7, SD 19.2) | digital transformation framework for public health services | factors affecting digital transformation, path and architecture of DT, perception of DT, identity of DT, satisfaction with DT, digital transformation framework for public health services | Not specified | data access, use, and disclosure | implement processes |  |
| Coarse-to-Fine Activity Annotation and Recognition Algorithm for Solitary Older Adults | Hu et al., 2020 | Experimental | 66.4±5.6 | automatic collection and labeling system , novel ADL algorithm | recognition accuracy of the algorithm | discovery and recognition of trivial ADLs | data collection  data aggregation | implement processes |  |
| Coupling simulation with machine learning: A hybrid approach for elderly discharge planning | Elbattah & Molloy, 2016 | Not specified | Not specified | hybrid approach (simulation model with machine learning) | an approach which addresses issues of discharge planning , population level questions, individual patient level questions | prediction on inpatient length of stay and discharge destination, prediction for healthcare resources | data aggregation | implement processes |  |
| Digital assessment of falls risk, frailty, and mobility impairment using wearable sensors | Greene et al., 2019 | Observational | 72.7 ± 10.7 years | novel digital assessment using wearable sensors | fall, fall risk, mobility impairment; algorithm performance | prediction of falls, frailty, and mobility impairment | data collection | implement processes |  |
| Early Detection of Parkinson’s Disease by Neural Network Models | Lin et al., 2022 | Experimental | 49-84 (with PD); 56-85 (control) | neural network models | accuracy/precision, specificity, sensitivity | early detection of PD | data collection | implement processes |  |
| Predicting Death or Disability after Surgery in the Older Adult | Shulman et al., 2023 | single-center prospective cohort study | 70–104 (78 ±6) | disability prediction model | model for disability and death prediction; model performance | prediction of death or disability | data collection | implement processes |  |
| The value of routinely collected data in evaluating home assessment and modification interventions to prevent falls in older people: systematic literature review | Daniels et al., 2021 | Systematic review | 60+ | use of routine collected data [i.e. EHRs] for home assessment and modification (HAM) interventions | Sources, type, and purpose of routine data | cost-effectiveness, hospitalization, incidence of falls, length of stay, care home admission, mortality | data collection | monitoring performance |  |
| Towards Data Governance for International Dementia Care Mapping (DCM). A Study Proposing DCM Data Management through a Data Warehousing Approach. | Khalid, 2011 | Not specified | Not specified | data care mapping through warehouse approach | DCM data warehouse | data governance framework | data access, use, and disclosure | implement processes |  |
| Unleashing the Power of Big Data for Alzheimer's Disease and Dementia Research | OECD, 2014 | Not specified | Not specified | big data | too many outcomes based on cited studies | AD and dementia research | data access, use, and disclosure | implement processes |  |
| Unobtrusive Monitoring to Detect Depression for Elderly With Chronic Illnesses | Kim et al., 2017 | Not specified | 69-90 | monitoring/sensing system | accuracy | ADL monitoring and depression detection | data collection | implement processes |  |
| Use of machine learning in geriatric clinical care for chronic diseases: a systematic literature review. | Choudhury et al., 2020 | Systematic review | 65 years and above | AI systems (machine learning) | current use of AI in geriatric care | geriatric care improvement among those with chronic diseases | data collection | implement processes |  |
| **People (n=3)** | | | | | | | | |  |
| Accountable Health Care Service Provisioning in the Cloud | Bernsmed, 2014 | Not specified | Not specified | M platform (cloud-based services) | roles, obligations, accountabilities; cloud service delivery chain | Not specified | data access, use, and disclosure | define accountability |  |
| Consent recommendations for research and international data sharing involving persons with dementia | Thorogood et al., 2018 | Scoping review | Not specified | consent for data sharing | recommendations (consent, decision making authority, support for decision making, planning in advance, representation, capacity assessment) | Not specified | data access, use, and disclosure | define accountability |  |
| Setting up a Governance Framework for Secondary Use of Routine Health Data in Nursing Homes: Development Study Using Qualitative Interviews. | Wieland-Jorna et al., 2023 | Not specified | Not specified | governance framework for routine health data | stakeholders’ perspectives. data governance framework | data governance | data access, use, and disclosure | implement processes |  |

Table S3. Research gaps on data governance in the healthcare of older persons.

|  | Data governance component, f | | |
| --- | --- | --- | --- |
|  | People | Process | Technology |
| N of cases | 3 | 19 | 35 |
| *Data life cycle* |  |  |  |
| Data collection | 0 | 12  ^[26], [34], [38-39], [41],[43]*, [44-49]^ | 29  ^[27-28], [31,36,40,55-66], [68-71], [73-76],^  ^[78-79], [81-82]^ |
| Data aggregation | 0 | 5  ^[34-25], [43]*, [50-51]^ | 0 |
| Data quality | 0 | 1  ^[43]*^ | 0 |
| Data storage | 0 | 1  ^[43]*^ | 1  ^[27]^ |
| Data protection | 0 | 1  ^[43]*^ | 0 |
| Data access use, and disclosure | 3  ^[32], [33], [37]^ | 5  ^[43]*, [39], [81],^  ^[42-53]^ | 5  ^[58], [67], [72], [77], [80]^ |
| Data retention and destruction | 0 | 0 | 0 |
| *Data governance function* |  |  |  |
| Define accountability | 2  ^[32], [33]^ | 0 | 1  ^[31]^ |
| Prioritize investment | 0 | 0 | 0 |
| Establish policy | 0 | 0 | 0 |
| Implement processes | 1  ^[37]^ | 16  ^[26], [35], [39,41-53]^ | 30  ^[54], [40], [55], [56-59],^  ^[60-73], [74-82]^ |
| Set standards | 0 | 2  ^[34], [38]^ | 0 |
| Manage risks | 0 | 0 | ^[36]^ |
| Monitor performance | 0 | ^[30]^ | ^[27], [28], [29]^ |

1. Multiple response (covered more than 1 data lifecycle); ^:^
2. [ ] Reference number
